# Spatial Bias in the Marine Fossil Record

**DOI:** 10.1371/journal.pone.0074470

**Published:** 2013-10-30

**Authors:** Daril A. Vilhena, Andrew B. Smith

**Affiliations:** 1 Department of Biology, University of Washington, Seattle, Washington, United States of America; 2 Department of Earth Sciences, The Natural History Museum, London, England, United Kingdom; University of Birmingham, United Kingdom

## Abstract

Inference of past and present global biodiversity requires enough global data to distinguish biological pattern from sampling artifact. Pertinently, many studies have exposed correlated relationships between richness and sampling in the fossil record, and methods to circumvent these biases have been proposed. Yet, these studies often ignore paleobiogeography, which is undeniably a critical component of ancient global diversity. Alarmingly, our global analysis of 481,613 marine fossils spread throughout the Phanerozoic reveals that where localities are and how intensively they have been sampled almost completely determines empirical spatial patterns of richness, suggesting no separation of biological pattern from sampling pattern. To overcome this, we analyze diversity using occurrence records drawn from two discrete paleolatitudinal bands which cover the bulk of the fossil data. After correcting the data for sampling bias, we find that these two bands have similar patterns of richness despite markedly different spatial coverage. Our findings suggest that i) long-term diversity trends result from large-scale tectonic evolution of the planet, ii) short-term diversity trends are region-specific, and iii) paleodiversity studies must constrain their analyses to well-sampled regions to uncover patterns not driven by sampling.

## Introduction

Patterns of biogeography, latitudinal diversity gradients, macroecology, and macroevolution result from biological processes constrained by the configurations of continents and earth processes [1]. Paleobiologists interested in the detection of these patterns and the processes that gave rise to them must first overcome unevenness and inconsistency of spatiotemporal sampling in the fossil record [2], [3]. This problem is manifested in various, often correlated ways: entire biogeographical regions without aragonitic shells [4], [5], uneven sampling of the latitudinal diversity gradient [6], inter and intra-regional variation in rock amount and quality [7]–[9], spatiotemporal differences in sampling effort and taxonomic identification [10]–[13], and cross-regional differences of preserved sedimentary environments and habitats [14], [15]. These biases can lead to erroneous results without proper precautions [16].

One important factor, largely overlooked to date, is the geographical distribution of sampling effort. As first noted by Allison & Briggs [17], if the spatial distribution of fossil taxa changes between time bins, how can we reject the hypothesis that observed changes in recorded diversity are due to shifts in spatial sampling patterns? A quantitative assessment of the spatial pattern of paleontological sampling and sampled taxonomic richness is therefore an essential first step for any large-scale paleobiodiversity analysis. Here we first document the biogeographical distribution of paleontological sampling and sampled taxonomic richness through the Phanerozoic and establish their strong covariance. We do this using the best available database of fossil occurrences, the Paleobiology Database (www.paleodb.org), which has been the preferred source of data for global Phanerozoic biodiversity analyses for over ten years [3], [13], [18]. Next, we demonstrate that the paleolatitudinal distribution of paleontological sampling and taxonomic richness does shift significantly over geological time, revealing time intervals of the rock record where geographical bias needs to be accounted for. Finally we apply modeling to see if we can establish how marine invertebrate diversity has changed over the Phanerozoic within fixed paleolatitudinal strips, controlling for sampling biases.

Overall, our study underscores the need to develop new subsampling approaches that can generate fossil datasets without spatiotemporal bias, which has ramifications for paleobiogeography, Phanerozoic diversity, and biostratigraphy.

## Methods

### 0.1 Data

The marine invertebrate groups with the best fossil records and which consequently are the most likely to have stable spatiotemporal sampling (anthozoans, brachiopods, echinoids, molluscs, and trilobites) were downloaded from the Paleobiology Database (PaleoDB). Subgenera were elevated to the genus level. From 481,613 occurrences across all but the earliest Cambrian (PaleoDB standardized 10 myr bins), we deduced the presence or absence of each genus for ten-degree paleolatitude strips from the paleolatitudes listed in the PaleoDB. To measure richness for each of the paleolatitude strips, we counted the number of these presences. Occurrences that were not constrained to one 10 myr bin were excluded. We chose paleolatitude because paleolongitude is less certain in deep time [19], and differential sampling of paleolatitudes is more likely to confound paleobiogeographical analyses [17]. Polar paleolatitude bins (90

S–80

S) and (80

N–90

N) were excluded from analyses to avoid edge effects.

Two measures of sampling bias were chosen, one measure for geographical breadth of sampling within a paleolatitude strip (to discriminate, for example, between paleolatitude strips where one marine shelf on one continent is sampled versus paleolatitude strips where many marine shelves on many continents are sampled), and one measure for collecting effort. We note that our intention is not to choose measures that correlate best with sampled richness, but rather to choose easily available proxies that capture the chief biases of sampled richness. These measures can be used in turn to produce estimates of richness that correct for these biases.

#### Measure of geographical breadth

**Figure 1:**
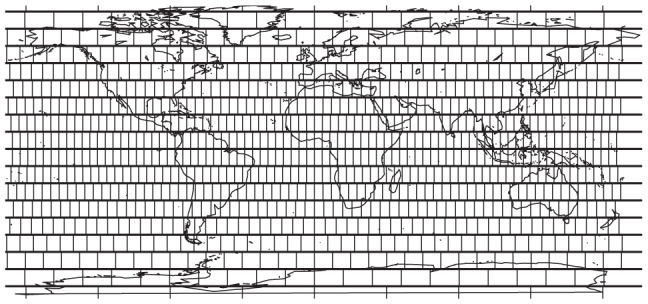
500,000 meter equal area gridding scheme for geographic coverage measure superimposed on a geographic map of the present day. This measure is equal to the number of equal area cells in a paleolatitude strip with fossil occurrences of our target taxa.

Geographical breadth of sampling within each paleolatitude strip (extent of sampling) was established by summing the number of equal area cells with fossil occurrences. Figure 1 shows our demarcation of equal area cells (500,000 meter wide cells). We note that this quantity is not normalized by the maximum number of equal area cells with epicontinental seas that could conceivably preserve fossils. These data would improve our measure but are not easily available at a global scale. This is unlikely to be a problem, however, because studies of the distribution of molluscan fauna have continually shown that provincial area does not correlate with the total richness of that area [20], [21], suggesting that latitude (associated with many key ecological factors such as temperatue and primary productivity) rather than occupiable area in a province (or area within a given latitude band) is a bigger driver of species richness.

#### Measure of collection effort

For each paleolatitude strip, we also counted the total number of collections with an identifier in the PaleoDB. Only collections that contained our target taxa were considered. We used this quantity as a measure of collecting effort. While collection sizes vary and cover different scales, these factors are unlikely to affect our global study of ten degree paleolatitude bands.

In principle, as sampling improves the spatial distribution of taxonomic richness should diverge from the spatial distributions of sampling proxies. Consider, for example, the astounding biodiversity of many taxonomic groups in the modern neotropics despite much more sampling in northern high latitudes.

### 0.2 Quantifying spatial shifts

In the results we demonstrate the strong covariance between our sampling proxies and sampled richness. Yet this is only a problem if paleolatitude strips that are well sampled in one time interval become poorly sampled in the next time interval. It is therefore critical to quantify spatial shifts in the recorded distribution of taxa in the fossil record, such as a shift from high to low latitude sampling. We measured the median paleolatitude of fossil occurrences for our target taxa. A Mann-Whitney U test was used to gauge whether the paleolatitudinal distribution of fossil occurrences for adjacent time intervals were statistically different.

### 0.3 Null model of richness

For comparison with empirical patterns, we model what time series of sampled diversity would result if we assumed true generic richness within paleolatitudinal strips is invariant over time and driven purely by sampling pattern following the approach of Smith & McGowan [22] and Lloyd [23]. To create a single sampling proxy, we multiplied the number of five by five degree grid cells within fixed paleolatitude strips sampled by the number of collections in those strips. Because our analysis focused only on single paleolatitude strips, demarcations based on meters were unnecessary. Note this sampling proxy captures the multiplicative relationship between geographic coverage and sampling effort (a ratio such as collections per grid cell would fail to predict richness – twice the grid cells and twice the collections would equal the same ratio despite producing more richness). The time series for the sampling proxy and richness within a particular paleolatitude band were first logged to remove the effects of outliers. Next the sampling proxy and taxon richness counts (drawn from the PaleoDB) were independently ordered from smallest to largest. The equation of the best-fitting model (a linear regression) to these data can then be applied to the rock record in its original time series. The difference between the expectation of this null model and the actual empirical count of sampled generic richness provides an estimate of whether diversity in a paleolatitudinal band is greater, less, or equal to what we would expect given the sampling proxy.

## Results

### 0.4 Determinants of the geographical distribution of richness

**Figure 2:**
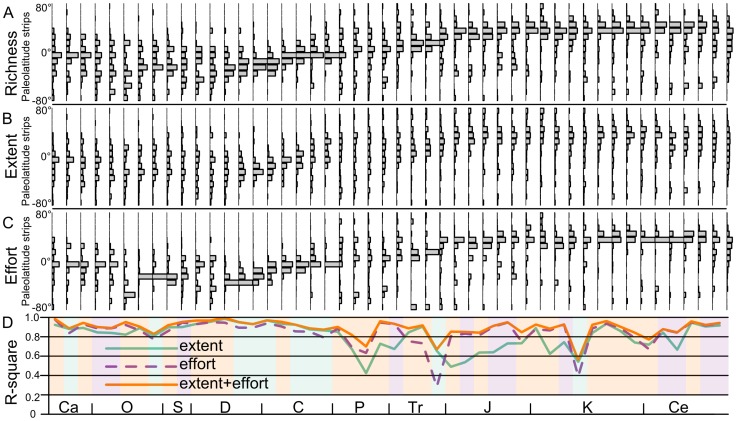
Patterns of richness and sampling proxies through the Phanerozoic. A) Distribution of genus richness across paleolatitude strips. B) Distribution of total equal area grid cells with at least one fossil locality recorded in the PaleoDB across paleolatitude strips. C) Distribution of faunal lists with collection IDs in the PaleoDB across paleolatitude strips. D) The percentage variation of richness in each paleolatitude strips explained by geographic coverage and sampling intensity in each of those paleolatitude strips. Note that the sampling proxies are not rendundant; parts of the Phanerozoic lack geographic coverage but have high sampling intensity and vice versa. Each interval is shaded by the color of the model with the lowest AIC score.

To visualize geographical richness patterns across the Phanerozoic, we plotted the distribution of generic richness next to the distribution of total sampled grid cells (extent) and collection effort (Figure 2A–C). To gauge how much of the spatial variation in richness (Figure 2A) is determined by the extent and intensity of geographical sampling (Figure 2B–C), we calculated the 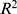 from a multiple regression analysis with richness as the response variable and the sampling proxies as the predictors. We logged each covariate to account for nonlinear relationships between richness, effort, and extent. We performed this analysis for each time interval. Figure 2D shows the resulting 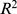 values for three models, sampling extent (number of sampled grid cells) and sampling effort alone (number of collections), and sampling extent and sampling effort combined.

All regressions were significant (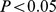). Extent alone has a mean 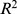 of 0.80. Extent alone predicts richness the worst in Permian 3 and Jurassic 1, dropping to 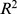 values of 0.42 and 0.49, respectively. Effort alone has a mean 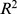 of 0.84 and predicts richness the worst in Triassic 4 and Cretaceous 4, dropping to 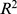 values of 0.28 and 0.39. However, this apparent inability of the sampling proxies to explain richness patterns for a few intervals is lost when the sampling proxies are combined as predictors. Extent and effort together never drop below an 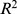 of 0.55, and the average 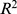 for this combined model is 0.89. AIC scores from these models reveal no systematic advantage of one model over another, with extent alone being the preferred model in 21% of the time intervals, effort alone being the preferred model in 25% of the time intervals, and the combined model being preferred for 54% of the time intervals.

Taken alone, extent and effort have systematically lower 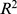 values through several intervals. However, the combined model is able to predict richness through these intervals, which suggests that intervals are differentially affected by extent and effort in the database. For example, intervals may be intensively sampled but lack geographic breadth, or contain geographic breadth but lack intensive sampling. This result underscores the need for more intensive sampling effort and geographic breadth in order to uncover global biological pattern.

### 0.5 Shifts in geographic coverage

**Figure 3:**
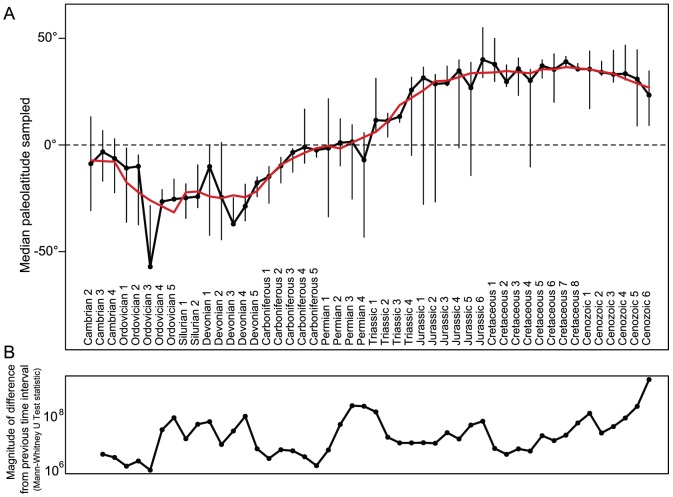
Spatial shifts in fossil occurrences through the Phanerozoic. A) The median latitude of fossil occurrences steadily rises through the Phanerozoic, but is punctuated by short-term noise and contractions and expansions of geographic coverage. Error bars indicate 25th and 75th quantiles, while the red line is a moving average across five points. B) The Mann-Whitney U test statistic plotted for each interval. A higher test-statistic corresponds to a more severe change in latitude. All transitions are statistically significant but vary in their effect size.

Our analysis reveals (i) a marked heterogeneity in paleolatitudinal coverage over time and (ii) marked shifts in paleolatitudinal coverage between time intervals. Figure 3A shows the median paleolatitude of fossil occurrences over the Phanerozoic, with the error bars reflecting the 25th and 75th percentile. To compare the long-term signal in the data with the median paleolatitude in each time interval, a red moving average line (5 points) is shown in Figure 3A. We tested for a difference between successive time intervals with a Mann-Whitney U test. All tests were significant (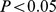) and Figure 3B shows the log value of the U statistic across the Phanerozoic. Repeated contractions and expansions of the error bars are evident, revealing repeated areas of the record where geographic coverage is expanded but then lost in the next time interval.

**Figure 4:**
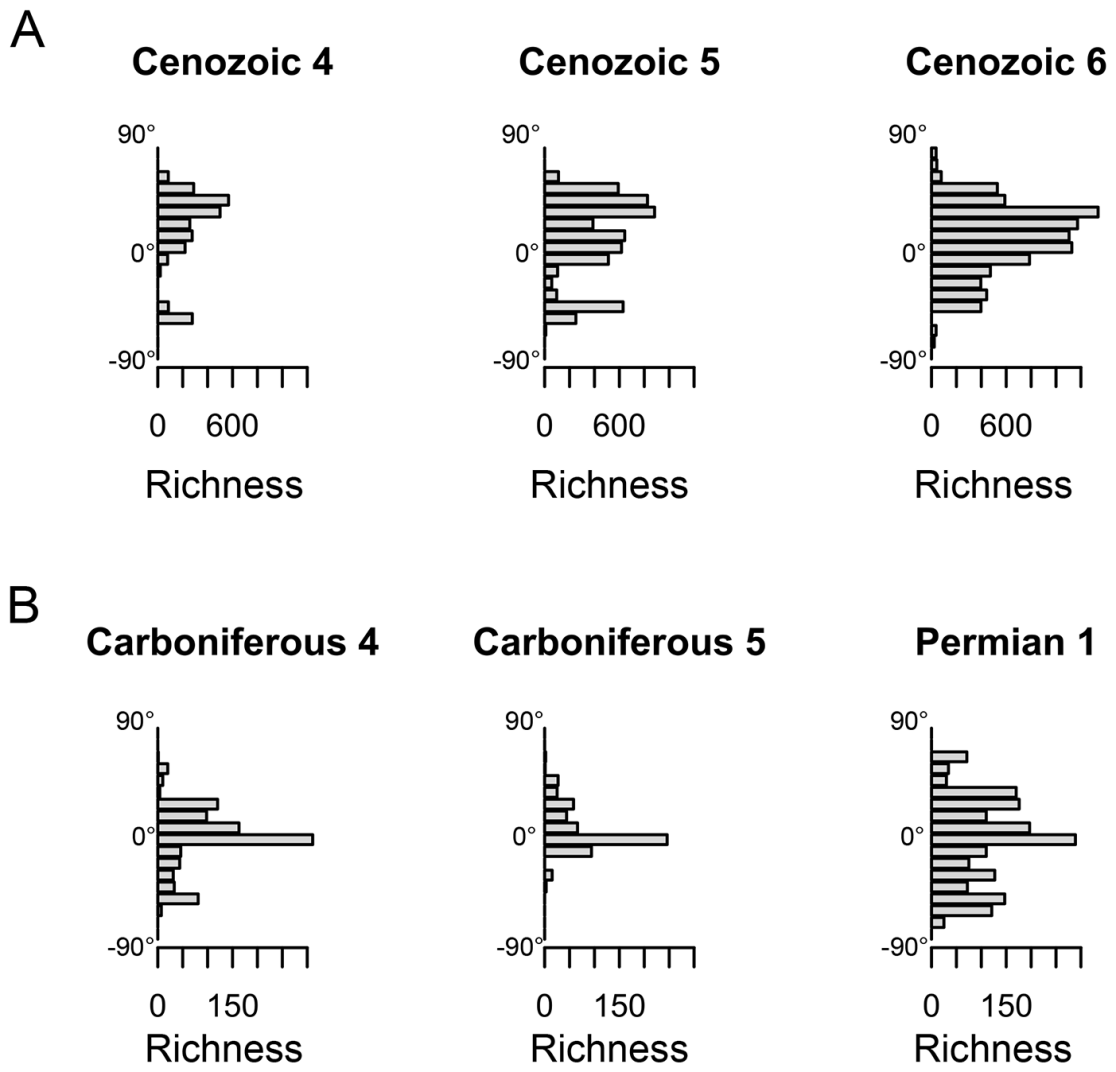
The distribution of genus richness across latitudes plotted for key intervals. A) For the last three Cenozoic time intervals (Cenozoic 4–6), Cenozoic 6 has more equitable sampling across latitudes than its predecessors. B) The Permo-Carboniferous boundary reflects a weakness in geographic coverage that biases estimates of global diversity inferred by subsampling.

Among the most drastic shifts includes the Middle Ordovician (Ordovician 2 to 3 and 3 to 4), with a shift in sampling from high to low latitudes (47 drop) and subsequent reversion (30 increase). The record remains comparatively stable until Devonian 1, with a 15 median increase. The record is volatile until the late Devonian, where the record appears to stably increase in median latitude, likely reflecting geologic signal rather than sampling bias. The PermoCarb boundary is exceptionally violent, with a vast expansion of geographical coverage in Permian 1. Geographic coverage is volatile until Jurassic 1, where the record remains mostly consistent. The remainder of the Mesozoic and Cenozoic is characteristic of well-sampled high latitude European and North American formations [7], though several intervals have sudden expansions of coverage that is subsequently lost (see Cretaceous 4). The last large shift is in the late Cenozoic (Cenozoic 6), with the recovery of Southern high latitude regions (Figure 4).

Some intervals have severe alterations of biogeographical representation. At the Permo-Carboniferous boundary, high latitudes (above 30 degrees) collectively increase richness by over 12-fold, while low latitude richness increases by just 2-fold.

### 0.6 Paleodiversity through time within fixed latitudinal strips

We examined spatial sampling and recorded genus richness through the Phanerozoic within two fixed paleolatitudinal bands, a paleotropical band from 10

S to 10

N and a paleotemperate band from 30

N to 50

**Figure 5:**
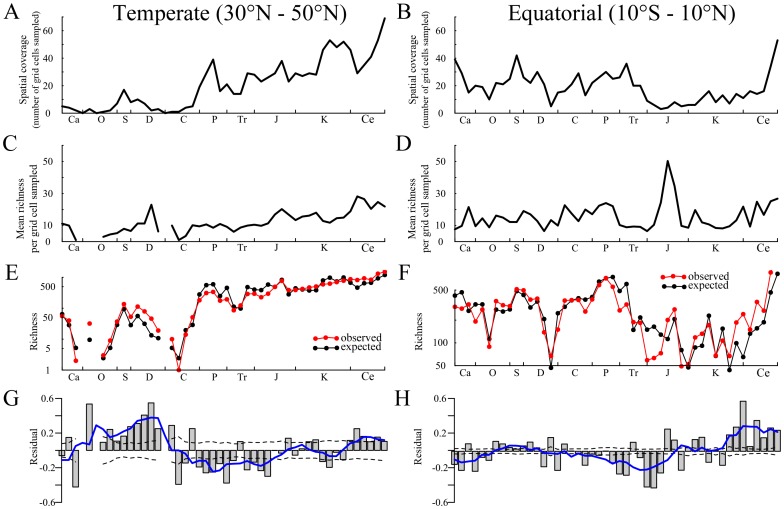
Diversity and sampling bias in latitude strips. A–B) Number of grid cells with sampled fossils for each time bin within two fixed paleolatiutudinal belts (temperate and equatorial). A gradual increase in sampled grid cells is evident in the temperate strip (A), while no such pattern is evident in the equatorial strip (B). C–D) Mean richness per sampled grid cell reveals no obvious pattern for faunas in the two paleolatitudinal belts. E–F) Null model that assumes biodiversity is driven purely by sampling (black) compared with observed genus richness (red). The null model explains the overall signal in the data, but select portions of the Phanerozoic deviate from the expectation. G–H) Plots show the difference between empirical richness and the expectation of the null model. Dashed bars indicate 99% confidence intervals for the null model. Overall, we find that the temperate and tropical faunas have similar trajectories despite markedly different trends in spatial sampling pattern over time.

N. Fig. 5A, B shows the relative spatial coverage attained in the two paleolatitudes. The paleotemperate latitude band shows a general rise in area sampled since the late Paleozoic, in marked contrast to the marked drop in spatial coverage in tropical latitudes from the Jurassic to the Neogene. The two time series are not correlated in the raw data: (Spearman rank correlation 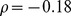, 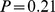) but after first differenced data show a marginally significant level (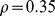, 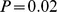). The differences between the two paleolatitudinal bands is largely explained by continental drift of well-studied areas from equatorial into temperate paleolatitudes over time.

To remove the effects of continental drift and spatial heterogeneity on Phanerozoic diversity estimates dominated by North American and European data we plotted the mean genus richness (number of genera divided by the number of equal area grids with records) over time in paleotropical and paleotemperate latitudes (Fig. 5C, D). While richness per unit area shows a progressive increase over time since the Carboniferous in temperate latitudes no such trend is apparent in tropical latitudes and the two time series show no correlation (Spearman rank correlation: raw data 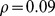, 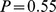; first differenced 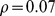, 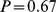). Furthermore the rise in temperate latitude richness is matched by a rise in the number of collections recorded in the database at these paleolatitudes (data not shown), as well studied continental blocks drift northwards over geological time. Changes in richness per unit area are strongly correlated with changes in sampling intensity per unit area. The lack of a common trend in richness per unit area over time between equatorial and temperate regions suggests sampling pattern is dominating the signal.

To discover what residual signal resides in these data that cannot be attributed to variation in sampling, we constructed models of Phanerozoic richness assuming true richness to be uniform over time and entirely driven by variation in sampling effort. The match between model and empirical data for both paleolatitude datasets is good but not perfect (Fig. 5E, F), with a significant proportion of the data left unexplained (Fig. 5G, H). Despite the very different spatial sampling records in these two bands (Fig. 5A, B), the residuals show similar long term trends: rising diversity from the Cambrian to mid Devonian, by a steady decline to a nadir in the Triassic (equatorial) to early Jurassic (temperate), followed by a rise to the Recent with short term downturns in the mid Cretaceous and Neogene (Fig. 5G, H). The two time series of residuals are positively correlated for raw data (Spearman rank correlation 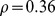, 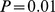), but not for first differenced data (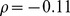, 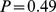).

## Discussion

Heterogeneity of the geological record makes it impossible to sample the fossil record uniformly over time. If we are to understand patterns of evolution then we need to first understand the nature of the problem and then develop methods that can compensate for such problems. Good progress has been made now to ensure that the fossil record that is preserved can be sampled fairly [13], [24]. But, before even the first fossil is collected, there is already an inbuilt bias to that record that needs to be taken into account if we are to interpret patterns of diversity correctly. So far consideration has only been given to how the macroarchitecture of the geological record affects our ability to sample fairly, either through controlling the amount of rock surviving from each time interval [7] or changing the proportional representation of environments that can be sampled [25]. What we show here is that there is also an inbuilt unfair sampling in terms of geographical coverage over time. Current methods that employ subsampling or rarefaction to correct for sampling irregularities assume that the area from which the data are drawn is uniform over time, which we now know is not true.

Two biases are chief candidates for driving spatial patterns in sampled richness: the distribution of sampling effort across latitudes, and the distribution of fossil localities across latitudes. In principle, the pattern of sampled richness should converge on the true, biological pattern of richness when sampling is equitable and sufficient across latitudes. However, before that point, the distribution of richness should mirror the geography of localities and effort. Limiting our analyses to the best-sampled groups within the largest macrofossil database available, we found that these biases explain the majority of the variation in richness for all time intervals. This suggests that the observed latitudinal richness for major marine animal groups, at least on a global scale, is a pattern that is majorly determined by sampling for the entire Phanerozoic.

Yet richness determined by sampling is not in itself a major problem so long as the geographic coverage of the data remains stable, because differences in sampling effort and rock amount between time bins could in principle be corrected for. However, spatiotemporal shifts in richness, driven by sampling, have the potential to profoundly influence analyses of biogeographical change and global biodiversity. For example, a change in relative sampling of distinct oceanic regions and climate zones [26], each with different levels of biodiversity and unique biotic compositions, could falsely give the impression of altered global diversity. We found a series of severe sampling shifts, with some time bins bearing little resemblance to the time bins before them (Figure 3A). The Paleobiology Database remains dominated by entries from Northern Hemisphere countries, particularly from Europe and North America (for example, 80% of Silurian records come from these two regions). This is reflected in our data, with long-term shifts in the relative dominance of sampling within different latitudinal bands created by the paleogeographical movement of well-studied regions over time. It is noteworthy that the median latitude in the sampling distribution steadily rises from the Cambrian to the latest Cenozoic (Figure 3A), and this has already been identified as a source of bias for diversity inference [17], [27].

Changes in sampled diversity that occur within blocks of time during which spatial shifts in richness are minimal clearly cannot be a result of differences in latitudinal representation in the database. However, where a change in diversity also coincides with a change in latitudinal sampling it is critical to test whether the latter could be driving the former. For example, we note that the last two periods of the Cenozoic, where diversity is perhaps rising, coincide with a rise in equitable sampling across latitudinal strips, with an overall high latitude richness increase of 3% and a low latitude richness increase of 118% (Figure 4A; above versus below 30 degrees). Our approach shows that it is impossible to reject the idea that the Neogene rise in global marine diversity is due to more equable latitudinal recording in the database, specifically in the tropics.

Another example is the major change in latitudinal sampling that occurs between the Late Carboniferous and Permian (Figure 4B). This coincides with anomalously low reported diversity for time interval Carboniferous 5 and high diversity for Permian 1 in sample standardized estimates of Phanerozoic diversity [3], [24], suggesting that uneven biogeographical sampling plays a large part in creating this. This is evident from our analysis of this boundary (Figure 3A), with a drastic increase in geographic coverage in Permian 1 (Figure 4B), and a 12-fold increase in high latitude richness.

Changes in the extent of epicontinental seas, driven by large-scale sea-level cycles or major tectonic events such as continental rifting or collision, can also affect the spatial distribution of fossiliferous marine deposits. So a common cause explanation [14], where the geographical distribution of marine fossiliferous rocks and marine diversity are both affected by the same driver, needs to be considered. Indeed, a recent study of genus richness and geographic area during the late Cretaceous [28] has clearly demonstrated a positive relationship between genus richness and geographic area in both epicontinental seas and ocean-facing coastlines. By looking at genus richness from fixed paleolatitudinal strips, our analysis draws data from multiple cratonic blocks, each with its own unique tectonic history, rock record [9], genus-area relationships [28], and idiosyncratic response to sea-level change [29]. Despite showing very different patterns of spatial sampling (Fig. 5A, B) and recorded genus richness through time (Fig. 5E, F, red line), the fossil records from equatorial and temperate paleolatitudinal strips reveal the same long-term trend in diversity after spatial sampling differences are accounted for (Fig. 5G, H). Diversity rises to the mid Devonian, falls gradually through the late Paleozoic and into the early Mesozoic before rising again to the Recent. While a similar long-term trend has been noted before [3], [3], [22], [24,30], our analysis provides the strongest evidence yet for it being a truly global biodiversity signal by showing it is replicated at different paleolatitudes. This strongly suggests that the long-term diversity trend is indeed the result of the large-scale tectonic evolution of the planet, following the first order Wilson cycles of continental accretion and dispersal as suggested previously [3], [22], [30].

On the other hand, small scale (stage-to-stage) shifts in genus richness (after accounting for spatial sampling differences) show no correlation between the two paleolatitudinal strips, suggesting that they were not responding to a common global driver but rather result from a sequence of region-specific events. This highlights that while a common cause explanation fits large-scale (100–200 myr cycle) patterns in the fossil record, the shorter (ca. 50–60 myr) cycles [31] are much more likely to be a reflection of region-specific changes in the original marine area [14], [28], [32] and the extent of any subsequent degradation of that rock record at outcrop [25].

In this paper we have shown that there is a strong geographical bias to the distribution of paleontological records and this needs to be taken into account in any assessment of biodiversity trends over geological time. Even in the best available database of fossil occurrences the fact that some time intervals are better sampled than others introduces a strong confounding effect on how we perceive global biodiversity to have changed over Phanerozoic time. In any biodiversity survey, sampling effort and recorded diversity will track each other initially but then start to diverge as sufficient records accumulate, until additional sampling adds no new records and they are effectively independent. We have shown that sampling effort in the fossil record still closely tracks recorded diversity and thus poses a severe problem for paleobiodiversity analysis. Yet, with the right techniques and approaches it is possible to tease out a global signal that is independent of spatial sampling biases. Spatial bias needs to be considered seriously in all future analyses of paleobiodiversity.
